# Pill Pushers or Partners in Progress: Should Pharmaceutical Companies Be Allowed to Sponsor Continuing Medical Education (CME)?

**DOI:** 10.7759/cureus.69156

**Published:** 2024-09-11

**Authors:** Satvik N Pai, Madhan Jeyaraman, Naveen Jeyaraman, Sankalp Yadav

**Affiliations:** 1 Orthopaedics, People's Education Society (PES) University Institute of Medical Sciences and Research, Bengaluru, IND; 2 Orthopaedics, South Texas Orthopedic Research Institute (STORI), Laredo, USA; 3 Clinical Research, Virginia Tech India, Dr. MGR Educational and Research Institute, Chennai, IND; 4 Orthopaedics, ACS Medical College and Hospital, Dr. MGR Educational and Research Institute, Chennai, IND; 5 Medicine, Shri Madan Lal Khurana Chest Clinic, New Delhi, IND

**Keywords:** conflict of interest, medical cme, medical conference, pharmaceutical companies, sponsorship

## Abstract

This article explores a timely and contentious issue within the Indian medical community: the relationship between doctors and pharmaceutical companies, particularly the sponsorship of continuing medical education (CME) events. Triggered by the National Medical Council's proposed guidelines in August 2023, which sought to ban such sponsorships, the article revisits the longstanding debate over potential conflicts of interest. While critics argue that these relationships could compromise medical ethics, the article questions whether this assumption holds up under scrutiny. It highlights the potential unintended consequences of a blanket ban, such as reduced opportunities for professional development, increased costs of healthcare services, and a slowdown in medical innovation. Furthermore, it offers a comparative analysis of global practices, revealing that no other country has imposed such a stringent ban, with other countries opting instead for regulated transparency. This one-of-a-kind article addresses a unique and underexplored topic in Indian medical literature, offering a nuanced perspective on how to balance ethical considerations with the need for continued medical advancement. Doing so calls for a more measured approach that preserves the integrity and progress of the medical profession.

## Editorial

The association between doctors and pharmaceutical companies has always been frowned upon by society, lawmakers, and also within large fractions of the medical fraternity. Any such association has been viewed with suspicion for such a long time now that we have almost stopped questioning why a relationship between two sections that are so vital to each other is considered unethical. This debate, however, has been revisited recently after the National Medical Council (NMC) released the latest guidelines for the professional conduct of doctors in August 2023. The new guidelines proposed to ban any sponsorship of medical conferences and continuing medical education (CME) sessions by pharmaceutical companies. It was also seeking to make it an offense for any medical professional to attend such a conference/CME that is sponsored by a pharmaceutical company. The guidelines saw criticism and backlash from a large number of medical practitioners and medical associations. While this issue was not the only clause criticized, it was one of the controversial points. The Indian Medical Association (IMA), the largest representative body of doctors in India, also opposed the new guidelines. Following discussions with representatives from the IMA, the NMC decided to temporarily repeal the new guidelines until further deliberations were undertaken. Although the new guidelines have been retracted for now, they have restarted the debate about the relationship between doctors and pharmaceutical companies.

What is the reasoning?

The relationship between doctors and pharmaceutical companies being discouraged is not something new. Even the Indian Medical Council (Professional Conduct, Etiquette, and Ethics) Regulations, which have been in force since 2002, outlawed medical professionals from receiving any sort of commissions and kickbacks [1]. In 2009, an amendment was made to these regulations to specifically address the Code of Conduct for doctors and professional associations of doctors in their relationship with the pharmaceutical and allied health sector industry [2]. The newly introduced clause 6.8 inhibited medical professionals from accepting any gifts, cash, or monetary payments from pharmaceutical or allied health industries. It even specified that medical professionals must not accept travel facilities or hospitality in terms of accommodations for any reason, be it vacation or to attend CMEs as delegates. The reasoning given behind this was that any association between a doctor and a pharmaceutical company would be unethical, as it would lead to doctors prescribing unnecessary medications or inappropriate medications for the sake of receiving compensation of some sort in return, and that the doctors would then not choose the right medication for the patient and instead make decisions based on which company they would receive gifts from. It was assumed that this would lead to an inherent conflict of interest between the patient's health and the monetary benefit for the doctor.

Is that really the case?

If, indeed, any association is adversely impacting patient health, it will definitely have to be discouraged. But are there any safeguards against this? Well, there is, in the basic fact that it is in the interest of the doctor as well as for the patient's health condition to improve. Suppose a patient's condition is not improving, in that case, it will not only be a negative for the doctor on humanitarian grounds but also because it would lead to the patient and attendees losing trust in the doctor and eventually to a decrease in the clientele for the doctor. So, if a doctor is prescribing certain medications that are not as effective purely for the reasons of kickbacks, his/her practice will face the consequences of such actions. No doctor under any circumstances is allowed to prescribe a substandard medication, as it is illegal for any such medication to be available for use that has not attained the required regulatory licenses. Considering that it is in the interest of the doctor for the patient's condition to improve for the sake of the patient and their own career, it is prudent for any doctor to choose the medications they advise wisely, valuing effectiveness, appropriateness, and affordability rather than any other factors.

Are we ignoring the impacts it can have on the other side?

The argument that this association should be banned does not really take into consideration the impact that such a ban would actually have. A huge proportion of the sponsorship for medical CMEs currently comes from pharmaceutical companies and manufacturers of medical devices. The first question people have raised regarding this is if these CMEs are even required, with many claiming that these CMEs are only meant for the merriment of doctors. Let us be clear: medical CMEs are absolutely required. CMEs are important tools in terms of updating knowledge for medical professionals. They facilitate networking, problem-solving, and collaboration. Their importance is highlighted by the fact that most state medical councils mandate doctors attend a certain number of CMEs every year in order to renew their medical licenses. CMEs play a vital role in sharing knowledge and the collective improvement in knowledge and skill of the medical fraternity, enabling them to provide better patient outcomes. Next, people have questioned whether CMEs require sponsorship. The answer is yes. The organization of a CME involves considerable costs. Apart from the major costs of venue and food, there are several other requirements: hospitality of speakers, prizes, event planners, and banners, to name a few. But what if the event is budgeted entirely on registration fees? That would be a nightmare for organizers. For one, the registration fees for the CMEs would have to be increased significantly compared to what it usually is currently when a major part of sponsorship comes from other sources. Second, it would put a real financial burden on the organizers to bring in the projected number of registrations for the event. If the registrations end up being less than estimated (likely considering the high registration fees), then that leaves the organizers in a real soup. Associations are likely to be apprehensive about taking such financial risks. If the doctors are the ones befitting, they should be willing to pay up the high registration costs, right? Fair point. But what is the likely outcome going to be? Doctors attend CMEs to improve their knowledge and skills and learn collectively. The ultimate aim is to improve patient outcomes. If the expenses of a doctor in the pursuit of their profession are going to increase, then it is going to be reflected in terms of an increase in medical services costs, outpatient department consultation fees, etc. We believe that if the rule banning sponsorship of CMEs by pharmaceutical companies comes into force, it will lead to a drastic decrease in the number of conferences/CMEs being conducted. Participation in CMEs will plummet drastically due to the high registration fees. The quality of CMEs and conferences would deteriorate because participation would become more about affordability than matter. Eventually, it will lead to increased healthcare costs and a decreased rate of advancement of medical knowledge among medical professionals.

Have we considered it from the point of view of pharmaceutical companies?

Many people have claimed that allowing pharmaceutical companies to sponsor these events would create bias among doctors for certain pharmaceutical companies and their products. Pharmaceutical companies and medical device companies choose to sponsor conferences in exchange for being allowed to keep stalls or hoardings to display their products. How is that really different from Pepsi running a commercial on television? Pharmaceutical companies should have the right to advertise their products. In India, the advertising of most medicines and medical devices is not allowed in public forums such as on television or in newspaper articles. There is merit in that law because it aims to prevent pharmaceutical companies from targeting the common man, who may not have the medical knowledge to understand the product or review its data. But what are the avenues left for pharmaceutical companies to be able to advertise their products? Imagine this. You have opened a pharmaceutical company and discovered a new drug that genuinely improves patient outcomes for a condition. You have the studies and results to prove it. But how do you then get the word out about it? You cannot advertise it on television or in newspapers. You cannot organize any event where doctors would be present to discuss its studies. You cannot even put up a hoarding at a conference for a doctor to see it. That is one drug and one medical condition. Now, imagine a scenario that would be applicable in the real world, where there are thousands of different medical conditions and drugs, with advances and innovations happening every year. Hence, the introduction of such a law will also make it difficult for pharmaceutical companies and manufacturers of medical devices to get the word out about any new products that can significantly improve patient outcomes.

A look at other countries

In order to gain a broader perspective on this issue and learn from other nations, we looked at the laws in relation to this aspect in other countries around the world. While many countries have regulations for the association between pharmaceutical companies and medical professionals, including sponsorship of CMEs and conferences, we found that no country has banned pharmaceutical companies from sponsoring CMEs. The country that comes closest to such a ban is France, with the Bertrand Act, which came into force in 2013 [3]. According to this law, it prohibits direct sponsorship by pharmaceutical companies. However, pharmaceutical companies can still provide financial support to independent organizations that conduct CME as long as these organizations maintain full control over the content. In the United States, sponsorship is allowed but must comply with the Accreditation Council for Continuing Medical Education (ACCME) standards for independence, and the financial relationship must be transparent under the Physician Payment Sunshine Act [4]. Other countries that have regulations for such a sponsorship include Australia, Canada, the United Kingdom, and the European Union [5]. These regulations generally aim to ensure that medical education remains independent and free from undue influence by the pharmaceutical industry, while still allowing for sponsorship.

So what should the law be?

We believe that a law banning pharmaceutical companies and manufacturers of medical devices from sponsoring CMEs and medical conferences will be a disservice to society. In the long run, it will lead to fewer CMEs, fewer participation in conferences, decreased knowledge sharing, increased doctors' charges, and eventually, a decrease in medical research, medical innovation, and medical advancement. The impact will be faced not only by medical professionals and pharmaceutical companies but also by patients and society. India would be the first country to introduce such an outright ban on sponsorship, and medical advancement in the country would face a major roadblock due to it. We must learn from other nations in having laws that regulate such an association, ensuring transparency and eliminating bias, rather than outright banning sponsorships. The way forward is collective progress, taking along all stakeholders and ensuring the sanctity of the profession, associations, and healthcare is maintained without hindering the massive progress Indian healthcare is making.

## References

[1] (2024). Code of Medical Ethics Regulations, 2002. https://www.nmc.org.in/rules-regulations/code-of-medical-ethics-regulations-2002/#:~:text=1%20A%20physician%20shall%20uphold,in%20accordance%20with%20its%20ideals.

[2] (2024). National Medical Commission. https://www.nmc.org.in/MCIRest/open/getDocument?path=/Documents/Public/Portal/LatestNews/press.pdf.

[3] (2024). The Bertrand Act - marketing is never going be the same again. https://www.baguette-trans.com/single-post/the-bertrand-act-marketing-is-never-going-be-the-same-again.

[4] (2024). ACCME policies. https://accme.org/rules/policies/.

[5] (2024). The EFPIA code. https://www.efpia.eu/relationships-code/the-efpia-code/.

